# Supplementary material for: Mutations in PCYT2 disrupt etherlipid biosynthesis and cause a complex hereditary spastic paraplegia
Source: Brain. 2019 Oct 22;142(11):3382–97. doi: 10.1093/brain/awz291 (PMC6821184; doi:10.1093/brain/awz291)
Supplement: awz291_Supplementary_Data [file awz291_supplementary_data.zip › awz291-Suppl_data/Supplementary_Data4.zip!/Supplementary information 4 - plasma lipidomics.zip!/Plasma lipidomics/Control vs Patient1/Statistical plots and data/TG[O] species/Boxplots all.pdf]

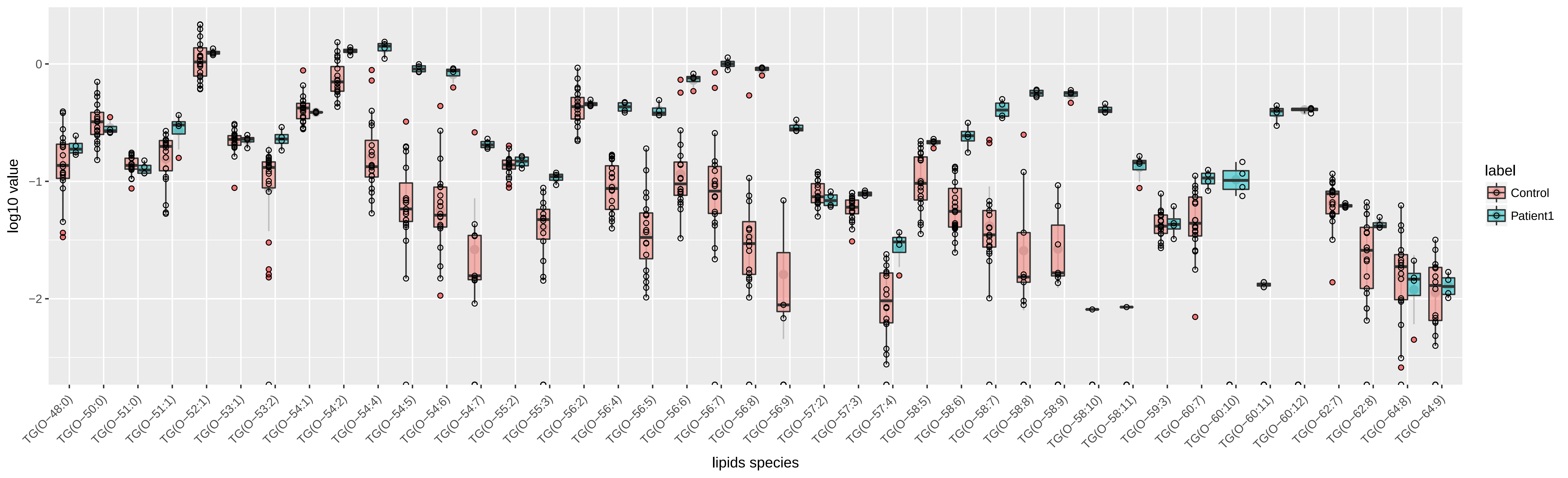

log10 value
0
−1
−2
lipids species
TG(O−48:0)
TG(O−50:0)
TG(O−51:0)
TG(O−51:1)
TG(O−52:1)
TG(O−53:1)
TG(O−53:2)
TG(O−54:1)
TG(O−54:2)
TG(O−54:4)
TG(O−54:5)
TG(O−54:6)
TG(O−54:7)
TG(O−55:2)
TG(O−55:3)
TG(O−56:2)
TG(O−56:4)
TG(O−56:5)
TG(O−56:6)
TG(O−56:7)
TG(O−56:8)
TG(O−56:9)
TG(O−57:2)
TG(O−57:3)
TG(O−57:4)
TG(O−58:5)
TG(O−58:6)
TG(O−58:7)
TG(O−58:8)
TG(O−58:9)
TG(O−58:10)
TG(O−58:11)
TG(O−59:3)
TG(O−60:7)
TG(O−60:10)
TG(O−60:11)
TG(O−60:12)
TG(O−62:7)
TG(O−62:8)
TG(O−64:8)
TG(O−64:9)
label
Control
Patient1
